# Patterns of multiple sclerosis presentation to the emergency department

**DOI:** 10.3389/fneur.2024.1395822

**Published:** 2024-04-26

**Authors:** Seraj Makkawi, Alaa Maglan, Osama Khojah, Faris Allaf, Saeed Alamoudi, Mohamed Eldigire Ahmed, Rawaf Alsharif, Meral Altayeb, Abdulrhman Alharthi, Ahmad Abulaban, Yaser Al Malik

**Affiliations:** ^1^College of Medicine, King Saud bin Abdulaziz University for Health Sciences, Jeddah, Saudi Arabia; ^2^King Abdullah International Medical Research Center, Jeddah, Saudi Arabia; ^3^Department of Neurosciences, Ministry of the National Guard-Health Affairs, Jeddah, Saudi Arabia; ^4^Fellowship and Residency Training Program, King Khaled Eye Specialist Hospital, Riyadh, Saudi Arabia; ^5^College of Basic Sciences and Health Professions, King Saud bin Abdulaziz University for Health Sciences, Jeddah, Saudi Arabia; ^6^Department of Neurology, Ministry of the National Guard-Health Affairs, Riyadh, Saudi Arabia; ^7^King Abdullah International Medical Research Center, Riyadh, Saudi Arabia; ^8^Department of Surgery, Ministry of the National Guard-Health Affairs, Jeddah, Saudi Arabia; ^9^College of Medicine, King Saud bin Abdulaziz University for Health Sciences, Riyadh, Saudi Arabia

**Keywords:** multiple sclerosis, emergency treatment, disease progression, disease modifying therapy, multiple sclerosis relapse

## Abstract

**Background:**

Multiple sclerosis (MS) patients are no strangers to the emergency department (ED) due to the relapsing and progressive nature of the disease and the associated complications. This study aimed to identify patterns of ED visits among patients diagnosed with MS, the underlying causes of these visits, and the factors associated with these visits.

**Methods:**

This was a single center retrospective cohort study which utilized a non-probability consecutive sampling technique to include all patients diagnosed with MS (471 patients) from March 2016 to October 2021 in King Abdulaziz Medical City, Jeddah, Saudi Arabia. ED visits were categorized as directly related to MS, indirectly related to MS, or unrelated to MS.

**Results:**

One in four people with MS visited the ED at least once with a total of 280 ED visits. Most ED visits were ones directly related to MS 43.6%, closely followed by unrelated to MS 41.1%, and then indirectly-related MS visits 15.4%. The most common presenting symptoms in directly-related MS visits were weakness 56.6% and numbness/tingling 56.6% followed by gait impairment 29.5%. Indirectly related to MS or unrelated to MS ED visits were commonly due to neurological 17.7% and gastrointestinal 17.1% causes. Using disease modifying therapy (DMT) was significantly associated with no ED visits (*p* < 0.001). The use of high-efficacy DMTs was significantly associated with no ED visits than using moderate efficacy DMT (*p* < 0.001). The use of B-cell depleting therapy (ocrelizumab and rituximab) was significantly associated with no visits to the ED than using any other DMT (*p* < 0.001). Evidence of brain atrophy on imaging was significantly associated with patients who presented to the ED ≥3 times (*p* = 0.006, UOR = 3.92).

**Conclusion:**

Due to the nature of the disease, many MS patients find themselves visiting the ED due to MS related and unrelated issues. These patients are not only required to be treated by neurologists but also by multiple disciplines. The use of high-efficacy DMTs and B-cell depleting therapy may reduce the total frequency of ED visits. Special attention should be paid to patients who have evidence of brain atrophy on imaging.

## 1 Introduction

Multiple sclerosis (MS) is a chronic neuroinflammatory disease that affects the central nervous system, T-cell-mediated inflammatory disease that affects the central nervous system (CNS) (1). MS is a growing concern in Saudi Arabia; from 2015 until 2018, a nationwide, multicenter MS registry in Saudi Arabia was established to describe the current epidemiology, disease patterns, and clinical characteristics of the disease (2). The registry included 20 hospitals from different regions in Saudi Arabia and collected data from 2,516 patients. It showed an overall prevalence of 40.40/100,000 of the total population and 61.95/100,00 among Saudi nationals (2). The data revealed a high prevalence of MS in Saudi Arabia, which was alarming and warranted immediate public health action (2). MS is characterized by episodes of limb weakness, numbness/tingling, visual impairment, poor coordination, and other neurologic deficits due to inflammation-induced demyelination, axon loss, and other neuronal injuries (1). MS activity is evidenced by new, enlarging, or enhancing lesions on magnetic resonance imaging (MRI), new clinical manifestations, and/or progression of preexisting symptoms (3). MS usually has a long preclinical period, with silent lesions on MRI at the time of clinical onset, and subtle deficits on clinical testing may be present years before symptoms appear (3). MS types include clinically isolated syndrome, relapsing–remitting, primary progressive, secondary progressive, and radiologically isolated syndrome (1). Approximately 80% of people with MS (pwMS) present, initially with the relapsing form of the disease (4). Exacerbations continue to occur throughout the relapsing–remitting stage of MS (4). These exacerbations and relapses force pwMS to seek acute care in the emergency department (ED) (5). Due to the chronic progressive and relapsing nature of the disease, pwMS are no strangers to the ED. Moreover, due to the systemic nature, non-specific disease course, and high level of disability, the presenting symptoms of pwMS are often versatile, depending on the type of lesions acquired and their activity level. In Saudi Arabia, there is a paucity of research on pwMS’ ED visit patterns and procedures. This study aims to identify patterns of ED visits among patients diagnosed with different types of MS and the underlying causes of these visits.

## 2 Methods

### 2.1 Patient selection

Our study cohort included 471 pwMS who were followed up in King Abdulaziz Medical City (KAMC), Jeddah, Saudi Arabia, from March 2016 to October 2021. KAMC is a tertiary care center with a specialized multiple sclerosis clinic. This retrospective cohort study utilized a non-probability consecutive sampling technique to include all patients diagnosed with MS. Patients were stratified based on whether they visited the ED. Those who visited the ED at least once were selected for further data collection. The total number of ED visits by pwMS was 280 visits. ED visits that occurred prior to establishing the MS diagnosis, or for the purposes of receiving medications (e.g., glucocorticoid injections), reviewing lab results, refilling medications, or testing for COVID-19 were all excluded.

### 2.2 Patient categorization

As was done by Oynhausen et al. (6), ED visits were categorized as directly related to MS (e.g., MS relapse, pseudo-relapse, worsening of chronic MS, or side effects of MS medications), indirectly related to MS [e.g., aspiration pneumonia due to bulbar weakness, urinary tract infection (UTI) due to bladder dysfunction, falls due to ataxia, and hardware malfunction (e.g., urinary catheter or a nasogastric tube)], or unrelated to MS (e.g., chronic obstructive pulmonary disease, myocardial infarction, and gastroenteritis). MS relapse was defined by the presence of new neurological symptoms or clinical worsening of pre-existing neurological symptoms lasting > 24 h in the absence of fever, heat exposure, or infection (5). Pseudo-relapse was defined as a temporary worsening of existing symptoms or return of symptoms secondary to a pro-inflammatory state such as an infection independent from MS (7). Patients who presented to the ED primarily due to temporary worsening of existing symptoms or return of symptoms (i.e., pseudo-relapse) fell under the directly related to MS category even if there were later found to have pro-inflammatory cause to their presentation. However, if they presented primarily due to an infection, then they would fall into indirectly related to MS or unrelated to MS category. Based on the clinical presentation and consulted specialty, patients who fell under the indirectly related or unrelated to MS categories were further divided based on the reason of the ED visit into gastrointestinal, pulmonic, obstetric and gynecologic, musculoskeletal, urologic, traumatic, ophthalmologic, vascular, immunologic, dental, cardiac, hematologic, dermatologic, otorhinolaryngologic, and psychiatric. Patients’ demographics, MS subtype, frequency of ED visits, date of MS diagnosis, and comorbidities were collected from all pwMS. The reason for the ED visit, the visit’s relation to MS, presenting symptoms, estimated expanded disability status scale (EDSS), brain and spine MRI findings, and treatment modality were collected from patients who visited the ED at least once.

### 2.3 Disease modifying therapies

The disease-modifying therapies (DMTs) used by patients in our institution included interferon beta, dimethyl fumarate, teriflunomide, glatiramer acetate, fingolimod, natalizumab, mitoxantrone, ocrelizumab, rituximab, alemtuzumab and azathioprine. DMTs were grouped into high-efficacy (HE) DMTs (natalizumab, ocrelizumab, rituximab, alemtuzumab, and mitoxantrone) and moderate-efficacy (ME) DMTs (interferon beta, dimethyl fumarate, teriflunomide, and fingolimod) (8).

### 2.4 Data analysis

The data was collected and entered using Microsoft Excel and then transferred and analyzed using the Statistical Package for Social Sciences (SPSS). The qualitative variables, such as gender, were presented as frequency and percentages. The quantitative variables, such as age and MS duration in years, were presented as mean ± standard deviation. The association between two categorical variables was assessed using the chi-square test. The association between numerical and categorical variables was assessed using the t-test. Bivariate analysis was used to assess the association between the frequency of ED visits with the other variables. The frequency of ED visits being more and equal or less than three were considered as a dependent variable. The cutoff value of three visits was determined based on the mean number of ED visits during the study period. Independent variables included the use of DMT, baseline EDSS, neurological symptoms, treatment at admission, investigations, disposition, and remission. Results were reported as an unadjusted odds ratio (UOR), and a *p*-value < 0.05 was accepted for significance.

### 2.5 Ethical consideration

This study was approved by the Institutional Review Board at King Abdullah International Medical Research Centre with the reference number JED-21–427780-46925, obtained on 30 March 2021.

## 3 Results

### 3.1 Patient demographics and characteristics

Of the 471 pwMS observed, 24.2% of them presented to the ED at least once. The total number of ED visits was 280 visits. Patients visiting the ED were mostly between the fourth and sixth decade of life, ranging in age from 15 to 74 years, with a gender predilection to females (M:F 1:1.95). The number of visits per patient ranged from one to 10, with an average of 2.6 visits per patient. The median duration of the disease was 8 years. More than three-quarters of patients were diagnosed with relapsing–remitting multiple sclerosis (RRMS). An ME DMT was used in 134 ED visits, an HE DMT was used in 34 visits, and no DMT was used in 112 ED visits. Patient characteristics are displayed in Table 1.

**Table 1:** Characteristics of multiple sclerosis patients.

Variable	Patients without an ED visit (*n* = 357)	Patients with an ED visit (*n* = 114)	Overall (*n* = 471)	*P*-value
**Gender**				
Male	118 (33.1%)	39 (34.2%)	157 (33.3%)	0.833
Female	239 (66.9%)	75 (65.8%)	314 (66.7%)	
Age	39.4 ± 11.4 years	40.6 ± 10.3 years	39.7 ± 10.5 years	0.296
BMI	26.9 ± 6.9 kg/m^2^	27.7 ± 6.4 kg/m^2^	27.1 ± 6.7 kg/m^2^	0.271
**Baseline EDSS**				
<6	308 (86.3%)	92 (80.7%)	400 (84.9%)	0.148
≥6	49 (13.7%)	22 (19.3%)	71 (15.1%)	
DMT	301 (84.3%)	96 (84.2%)	397 (84.3%)	0.979
**MS subtype**				
CIS	7 (2%)	5 (4.4%)	12 (2.5%)	0.223^*^
RRMS	294 (82.4%)	96 (84.2%)	390 (82.8%)	
SPMS	16 (4.5%)	11 (9.6%)	27 (5.7%)	
PPMS	9 (2.5%)	2 (1.8%)	11 (2.4%)	
RIS	1 (0.2%)	0 (0%)	1 (0.2%)	

ED, Emergency Department; BMI, Body Mass Index; EDSS, Expanded Disability Status Scale; DMT, Disease Modifying Therapy; CIS, Clinically Isolated Syndrome; RRMS, Relapsing–Remitting MS; SPMS, Secondary Progressive MS; PPMS, Primary Progressive MS. *p*-value < 0.05 is significant. ^*^There were 30 patients who were unclassified; none of them visited the ED.

No significant association was found between the frequency of ED visits and age, gender, body mass index (BMI), baseline EDSS, or MS subtype. Moreover, there was no significant difference between RRMS and progressive MS [secondary progressive MS (SPMS) and primary progressive MS (PPMS)] patients and presenting to the ED or not (*p* = 0.195). Chronic hypertension and dyslipidemia were significantly associated with frequenting the ED at least once (*p* = 0.041) and (*p* = 0.047), respectively. ED visits varied based on the day of the week, as more people visited the ED on Saturday (16.1%), Sunday (17.8%), and Monday (17.8%) than on Tuesday (12.9%), Wednesday (15%), Thursday (11.8%), and Friday (8.6%).

### 3.2 Chief complaint at the emergency department

**Figure 1:**
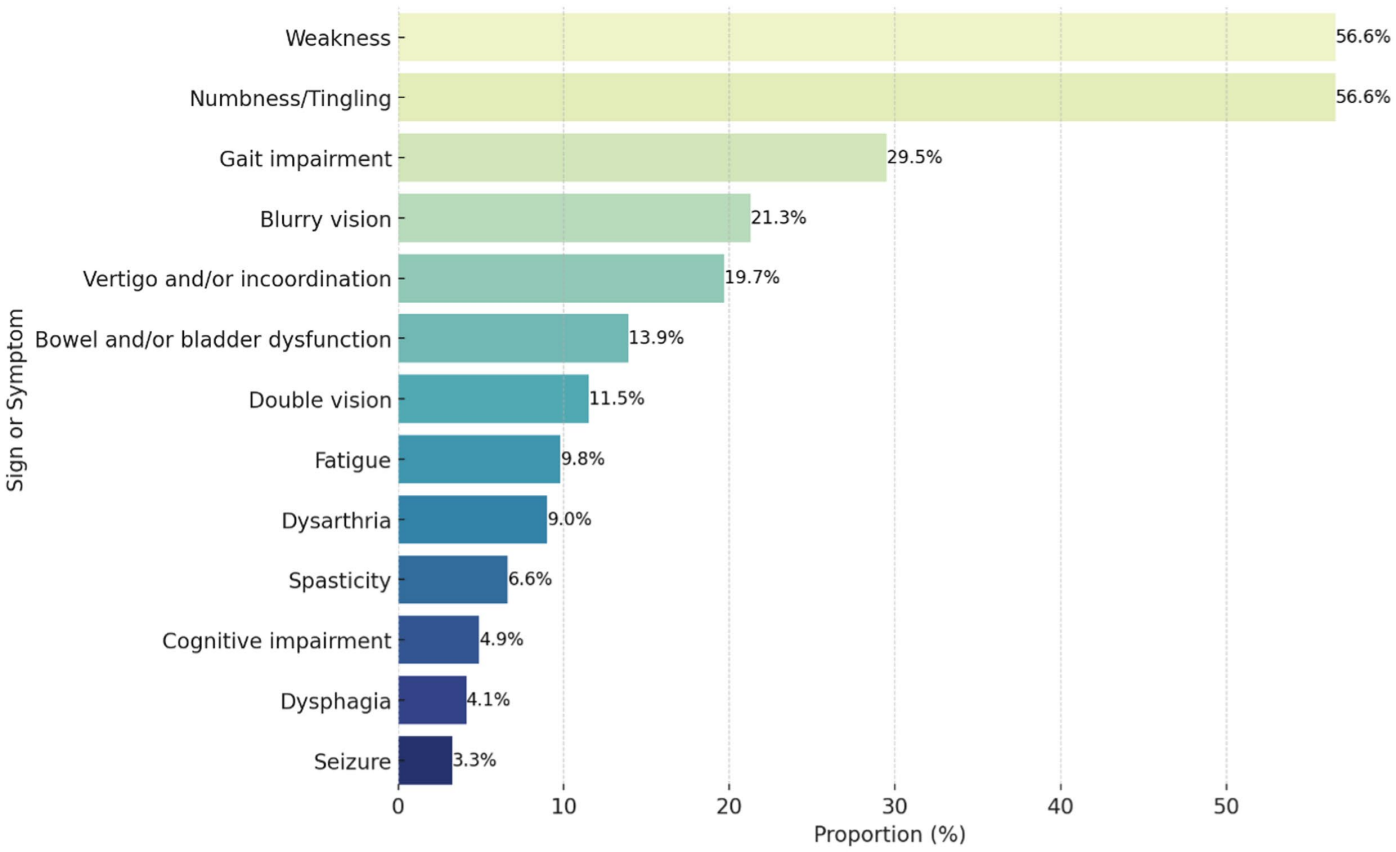
Proportion of clinical signs and symptoms in patients presenting with a directly related to multiple sclerosis emergency department visit (*n* = 122).

**Figure 2:**
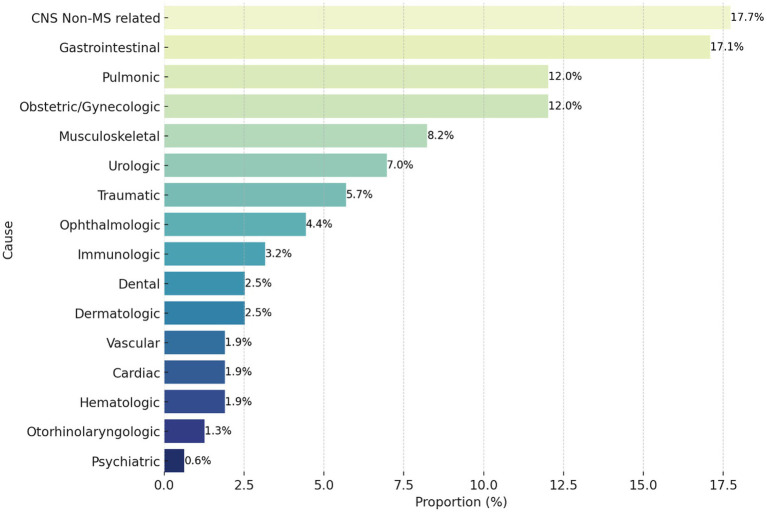
Proportion of causes for emergency department visits indirectly related/unrelated to multiple sclerosis (*n* = 158). CNS, central nervous system; MS, multiple sclerosis.

Most ED visits were directly related to MS 43.6%, followed by unrelated to MS 41.1%, and then indirectly-related MS visits 15.4%. Out of the 122 visits directly related to MS, relapse was found in 79.5% of visits, pseudo-relapse in 13.1% progression in 4.9%, and fluctuation in 2.5% of visits. The most common presenting symptoms in directly-related MS visits were weakness 56.6% and numbness/tingling 56.6% followed by gait impairment 29.5% (Figure 1). The majority of patients who presented with CNS non-MS causes presented with headache. In general, almost half 46.4% of ED visits by pwMS were due to non-neurological causes. Causes of ED visits that were indirectly related to MS or unrelated to MS are represented in Figure 2. A significant association between weakness (*p* = 0.032) or spasticity (*p* = 0.004) and presenting to the ED three times or more was found with an unadjusted odds ratio of 2.42 and 8.22, respectively.

### 3.3 The effect of DMT on ED visits

Out of all pwMS who visited KAMC during the 6 years, 84.3% were DMT users. Of all ED visits by pwMS, 43.6% were directly related to MS. Of those visits, 69.3% occurred while patients were active DMT users. In those users, interferon beta 36.7% fingolimod 30.4%, and natalizumab 16.5% were the most used (Table 2). The mere use of DMTs was significantly associated with no ED visits (*p* < 0.001) when compared to not using a DMT. Further analysis showed that the use of HE DMTs was significantly associated with no ED visits when compared to no DMT use and the use of ME DMT; (*p* < 0.001) and (*p* < 0.001), respectively. Moreover, the use of ME DMTs was significantly associated with no ED visits when compared to no DMT use (*p* < 0.001). The use of B-cell depleting therapy (ocrelizumab and rituximab) was significantly associated with no ED visits than when any other DMT was used (*p* < 0.001). Table 3 describes the use of DMT between patients without ED visits and the DMTs used in each ED visit.

**Table 2:** Disease modifying therapy used by patients visiting the emergency department based on the visit’s relation to multiple sclerosis.

Disease modifying therapy	Directly related to MS visit (*n* = 122)	Indirectly related to MS visit (*n* = 43)	Unrelated to MS visit (*n* = 115)	Total (*n* = 280)
Interferon beta	29 (23.8%)	9 (20.9%)	31 (27%)	69 (24.6%)
Fingolimod	24 (19.7%)	3 (7%)	11 (9.6%)	38 (13.6%)
Natalizumab	13 (10.7%)	6 (14%)	2 (1.7%)	21 (7.5%)
Dimethyl fumarate	5 (4.1%)	2 (4.7%)	8 (7%)	15 (5.4%)
Teriflunomide	4 (3.3%)	2 (4.7%)	6 (5.2%)	12 (4.3%)
Ocrelizumab/rituximab	2 (1.6%)	1 (2.3%)	7 (6.1%)	10 (3.6%)
Alemtuzumab	2 (1.6%)	0 (0%)	1 (0.9%)	3 (1.1%)
No DMT used	43 (35.2%)	20 (46.5%)	49 (42.6%)	112 (40%)

**Table 3:** Disease modifying therapy effect on the frequency of emergency department visits.

Variable	No ED visits	ED visits^*^	*P*-value
DMT non-user	56 (33.3%)	112 (66.7%)	<0.001
DMT user	301 (64.2%)	168 (35.8%)	
DMT non-user	56 (33.3%)	112 (667%)	<0.001
ME DMT user	155 (53.6%)	134 (46.4%)	
DMT non-user	56 (33.3%)	112 (667%)	<0.001
HE DMT user	146 (81.2%)	34 (18.9%)	
ME DMT user	155 (53.6%)	134 (46.4%)	<0.001
HE DMT user	146 (81.2%)	34 (18.9%)	
B-cell depleting therapy user	107 (91.5%)	10 (8.5%)	<0.001
Other DMTs user	194 (55.1%)	158 (55.9%)	

ED, Emergency Department; DMT, Disease modifying therapy; ME, Moderate efficacy; HE, High Efficacy. ^*^Total number of ED visits was 280 visit, ME DMT was used in 134 ED visits, HE DMT was used in 34 visits, no DMT was used in 112 ED visits, and B-cell depleting therapy DMT was used in 10 ED visits. *p*-value < 0.05 is significant.

### 3.4 Investigations for patients with directly related to MS visit visits to the ED

In patients who presented to the ED due to a directly related to MS visit, brain and spine MRIs were performed within 1 year of the ED visit in 72.1% of visits. Of those visits, one or more new or enlarging lesions was found in 63.6% and enhancing lesions in 13.6%. Of the 56 patients with a new or enlarging lesions, about 33.9% of people had one new lesion, 16.1% had two new lesions, and 50% had three or more new lesions. These lesions were most commonly found in the cortical/juxtacortical area 66.1%, followed by infratentorial area 51.8%, spinal cord 33.9%, and periventricular area 30.4%. A significant association was found between MRI utilization and patients who had fewer than three ED visits (*p* = 0.023; UOR = 0.402). Out of 77 baseline MRIs, one or more spinal cord lesions in was found in 50.6% of patient visits, extensive disease in 24.7%, and brain atrophy in 35.1% of patient visits. Brain atrophy was significantly associated with patients who presented to the ED ≥3 times (*p* = 0.006, UOR = 3.92). Urinalysis was performed in 22.1% of ED visits directly related to MS, mainly for patients who had urinary symptoms at presentation to the ED. Only 25.9% of them had a documented positive nitrate and WBC result, and 11.1% had a positive culture. UTIs accounted for 25% of pseudo-relapses.

### 3.5 Management and outcomes

Of the 97 patient visits due to MS relapse, 39.3% of them led to admission to the neurology department, and one patient was admitted under another specialty. While 59.8% of patient visits led discharge from the ED, as patients preferred to come to the hospital to receive intravenous pulse methylprednisolone every day for 5 days without admission. For the patients diagnosed with relapse, 93.4% of the patients received glucocorticoids, and three of those who did not show clinical improvement after receiving pulse steroids were treated with plasmapheresis. Only 4.9% of patients who had relapsed with non-disabling symptoms did not receive steroids, and they were given a close follow-up appointment in the clinic. Upon discharge, 72.6% of patients had complete remission, 19.8% had partial remission, and only 7.5% had no remission of their symptoms.

## 4 Discussion

In this study of 471 pwMS, one in four patients visited our ED at least once during the study period. The reason for their visits varied widely, though were predominantly neurological in nature. We also described the types of presenting symptoms with regards to their relation to MS, with the directly related ones being the most common reason warranting a visit to the ED.

During the study period, almost one in four pwMS visited the ED at least once. This highlights the importance of prepared emergency departments, emergency physicians, and neurologists. Graf et al. (9) reported that out of 15,350 pwMS, 8,603 (56%) patients visited the ED at least once; reaching a total of 37,072 ED visits. One study in the US showed that 61.2% of pwMS were diagnosed during an ED visit, and that percentage rose to 73.5% within a week of the visit (9). Another study showed that pwMS’ visits to the ED post-diagnosis within 3 years can range from one visit to 15 visits, with an average of 2.5 visits per patient which is similar to our findings (6). Of those visits, only 25.5% were neurological complaints, the majority of which were for weakness 46.2%, and the other 74.5% of visits were for non-neurological reasons (6). In our study, almost half of the presentations to the ED by pwMS were for non-neurological reasons. This result is comparable to Abboud et al., who reported a rate of 44.8% of non-neurological reasons for ED visits over 1 year, but contrasts with the 74.5% in Oynhausen et al. (6, 10). Weakness and numbness/tingling were the most common neurological presentations but were not directly related to the number of visits over the study period. Weakness was also the most common presentation in Oynhausen et al. (6), while numbness/tingling was reported at a lower presentation rate (9%). The severity of the condition as calculated by the EDSS score did not influence the number of times patients visited the ED; however, the type of presentations did. The two neurological symptoms associated with increased frequency of ED visits were weakness and spasticity, outlining an area for further investigation regarding the outpatient management and education of pwMS regarding these symptoms.

Using commercial insurance and Medicare Advantage claims, Zhu et al. (11) conducted a US study which included 25,932 pwMS and found that 48.9% of patients were on a DMT. In contrast, 84.3% of our patients were on a DMT. High utilization of DMTs in our population could be due to the fact that access to healthcare and medications in Saudi Arabia is for free and does not apply any restrictions, whereas the annual cost for DMTs in pwMS in the US exceeds 70,000$ (12). Zhu et al. (11) also found that patients on HE or ME DMTs had more relapses than those left untreated. There are conflicting reports in literature as it pertains to the effect of DMT on ED visits; One study in British Columbia, Canada, investigated the use of DMT and its relation to the frequency of visits to the ED and found similar numbers on both arms of comparison as 58.0% of DMT users visited the ED whereas 55.6% of DMTs non-users visited the ED (9). Mazibrada et al. (13) found that the utilization of emergency services rose from 0.5% prior to using fingolimod to 5.3% after using fingolimod in patients with RRMS. Akin to our results, a study conducted by Sanchirico et al. (14) found that patients on DMT had less ED visits than those who were not on DMT. Also, Bonafede et al. (15) found that patients on natalizumab had significant reduction in health care utilization. However, we not only found that DMT use did affect ED visits, but also that different types of DMTs affected them differently, with B-cell depleting therapy playing a protective role. These reports in the literature which find their patients having more relapses on DMTs might be due to the fact that many of the DMT non-users have a stable disease. This finding should be a factor for consideration when DMTs are initially prescribed. Also it opens the door for a better look into the effects of DMT on the severity of symptoms when visiting the ED, an angle not looked into by our study due to the limitations of retrospective research.

The likelihood of patients presenting with a neurological symptom undergoing an MRI within 1 year of their visit was inversely related to the number of visits done by the patients. Patients with three or fewer visits were significantly more likely to undergo an MRI than those with a higher number of visits. This could be because physicians may have a lower threshold for those patients who do not frequently present. Our results also revealed that only 22.1% of MS visits to the ED included a urinalysis, with no correlation between the number of visits and the likelihood of it being performed. Our study demonstrated that the frequency at which urinalysis is being performed is far less than necessary. It was most commonly utilized in patients who had urinary symptoms. However, a urinalysis should be performed on all pwMS presenting to the ED to assess the nature of the visit. Urinalysis is an important and relatively inexpensive screening device for UTIs, a common non-neurological complication of MS that requires swift diagnosis and treatment, and a simple method to differentiate true relapses of MS from pseudo-relapses.

MS is a chronic inflammatory demyelinating condition of the CNS and a leading cause of disability in young adults (6). A female predilection in our study is concordant with local studies, including the Saudi registry (2). This observation extends beyond regional boundaries and is documented in western populations, as seen in a study conducted in Mount Sinai in the United States, where the proportion of females was 69% (6). The mean age in our sample is almost 40 years, which is comparable to the Saudi registry in which 70% were no older than 40 years old (2). Other regions have reported a slightly younger age, which is consistent with the known peak age of incidence in MS worldwide (16–18). Obesity is regarded as a risk factor for developing MS, yet there is a paucity of data on average BMI in pwMS; among our sample, the mean BMI was 27.1 kg/m^2^ (19). One of the challenges in the categorization of MS subtypes is the distinction between RRMS and SPMS. This is because SPMS is often diagnosed retrospectively, after a patient who was initially diagnosed with RRMS experiences a progressive decline in neurological function without relapses. Additionally, there are no clear criteria for defining the transition from RRMS to SPMS. These factors can make it difficult to accurately determine the prevalence of SPMS, and it may have underestimated the prevalence of SPMS in our cohort (20). The EDSS was used to evaluate patients’ disease status at each visit, and most subjects with and without ED visits had an EDSS<6 (80.7 and 86.3%, respectively), which was not significantly related to the frequency of ED visits (*p*-value = 0.148). In contrast, a US study found that 55.4% of subjects had severe MS (defined as EDSS ≥ 6) (6). The unequal distribution between the two EDSS groups may have hindered our ability to find a significant correlation between EDSS estimations and the frequency of ED visits. Interestingly, those who had severe MS were three times more likely to present to the ED with complaints indirectly related to MS (e.g., pneumonia, UTIs, falls) compared to those with mild and moderate MS (defined as EDSS < 6) (6). On the other hand, mild and moderate disease had more directly related to MS complaints as the cause of their visits, such as MS relapse or disease progression (6). This highlights the importance of training physicians to identify MS-related complaints—whether direct or indirect—to provide optimum care given the diverse presentation of the disease.

MS is commonly associated with several comorbidities. A study found that almost 77% of pwMS had at least one comorbid condition (21). Metabolic comorbidities are frequently encountered in pwMS, and their management should be optimized to avoid adverse effects on functional ability and survival (19, 21). Our subjects demonstrated a striking disparity with regard to the proportion of metabolic comorbidities compared Marrie et al. (21), who reported associated comorbidities in patients with MS. We found only 7% of our sample were hypertensive as compared to 30% in the cohort of Marrie et al. (21). Dyslipidemia was even less common among our patients at 2.65% compared to 37% in other subjects (21). No previous studies have demonstrated a tendency of patients with MS and certain comorbidities to suffer more frequent ED visits. We found that pwMS with hypertension or dyslipidemia had a significantly higher frequency of ED visits. It should be noted that these comorbid conditions can have deleterious effects on quality of life and have even been shown to be related to a decline in cognitive function among pwMS (17, 22). A strong inverse correlation was found between total serum cholesterol levels as well as LDL levels and significantly lower scores in multiple cognitive function scales among pwMS with dyslipidemia (17, 22).

### 4.1 Limitations

This study may be limited by its single-center and retrospective design. It is important to mention the location of KAMC as it lies on the periphery of the city of Jeddah. In addition, KAMC is a tertiary center that serves patients who come from all over the western region of Saudi Arabia. This makes it inconvenient for some patients to visit the hospital for presentations they deem undeserving of the trip. When the outbreak of COVID-19 occurred, a total curfew was implemented from 23rd of March 2020 to 13th of June 2020. Although there was an exception to medical emergencies, this curfew may have made it difficult for patients to reach the needed help. Finally, We were not able to independently assess and exclude potential false-positive diagnoses.

## 5 Conclusion

This study found that approximately one-quarter of pwMS presented to the ED at least once during the study period. The causes of ED visits were almost equal, with half being neurological and half being non-neurological. The most common chief complaints in patients presenting with a directly related to multiple sclerosis ED visits were weakness, numbness/tingling, and gait impairment. The type of DMT used significantly affected the likelihood of visiting the ED, with patients on B-cell depleting therapy being less likely to present. Hypertension and dyslipidemia were significantly associated with more ED visits. Patients with brain atrophy were significantly associated with presenting to the ED three times or more. Clinicians should be aware of the increased risk of hypertension, dyslipidemia, and brain atrophy in pwMS. These conditions can all increase the risk of ED visits, and therefore clinicians should be vigilant in monitoring for and managing these conditions in pwMS. The study highlights the need for further research on MS emergency needs and the optimization of their management. Future studies should focus on developing and evaluating strategies to reduce the frequency and severity of ED visits in pwMS.

## Data availability statement

The raw data supporting the conclusions of this article will be made available by the authors, without undue reservation.

## Ethics statement

The studies involving humans were approved by the Institutional Review Board at King Abdullah International Medical Research Centre (Reference number: JED-21–427780-46925). The studies were conducted in accordance with the local legislation and institutional requirements. Written informed consent for participation was not required from the participants or the participants' legal guardians/next of kin in accordance with the national legislation and institutional requirements.

## Author contributions

SM: Conceptualization, Methodology, Supervision, Writing – original draft, Writing – review & editing, Validation. AM: Data curation, Formal analysis, Investigation, Writing – original draft, Writing – review & editing. OK: Conceptualization, Data curation, Formal analysis, Investigation, Methodology, Writing – original draft, Writing – review & editing. FA: Investigation, Writing – original draft, Writing – review & editing. SA: Investigation, Writing – original draft, Writing – review & editing. MAh: Formal analysis, Methodology, Writing – original draft, Writing – review & editing. RA: Investigation, Writing – original draft, Writing – review & editing. MAl: Investigation, Writing – original draft, Writing – review & editing. AAl: Investigation, Writing – original draft, Writing – review & editing. AAb: Conceptualization, Investigation, Methodology, Supervision, Writing – original draft, Writing – review & editing. YM: Investigation, Methodology, Supervision, Writing – original draft, Writing – review & editing.
